# Standard Bismuth Quadruple Therapy versus Concomitant Therapy for the First-Line Treatment of *Helicobacter pylori* Infection: A Systematic Review and Meta-Analysis of Randomized Controlled Trials

**DOI:** 10.3390/jcm12093258

**Published:** 2023-05-03

**Authors:** Rocco Maurizio Zagari, Elton Dajti, Anna Cominardi, Leonardo Frazzoni, Lorenzo Fuccio, Leonardo Henry Eusebi, Amanda Vestito, Andrea Lisotti, Giuseppe Galloro, Marco Romano, Franco Bazzoli

**Affiliations:** 1 IRCCS Azienda Ospedaliero-Universitaria di Bologna, Sant’Orsola Hospital, 40138 Bologna, Italy; elton.dajti2@unibo.it (E.D.); leonardo.frazzoni@gmail.com (L.F.); lorenzo.fuccio3@unibo.it (L.F.); leonardo.eusebi@unibo.it (L.H.E.); amanda.vestito@gmail.com (A.V.); 2 Department of Medical and Surgical Sciences, University of Bologna, 40138 Bologna, Italy; franco.bazzoli@unibo.it; 3 Gastroenterology and Hepatology Unit, Piacenza Hospital, 29121 Piacenza, Italy; annacomi26@gmail.com; 4 Gastroenterology Unit, Hospital of Imola, 40026 Imola, Italy; lisotti.andrea@gmail.com; 5 Surgical Endoscopy Unit, Department of Clinical Medicine and Surgery, Federico II University, 80138 Naples, Italy; giuseppe.galloro@unina.it; 6 Hepatogastroenterology and Digestive Endoscopy Unit, Department of Precision Medicine, University of Campania “Luigi Vanvitelli”, 80138 Naples, Italy; marco.romano@unicampania.it

**Keywords:** *Helicobacter pylori*, bismuth quadruple therapy, concomitant therapy, first-line treatment

## Abstract

(1) Background: Whether standard bismuth quadruple therapy (BQT) is superior to concomitant therapy for the first-line treatment of *Helicobacter (H.) pylori* infection is unclear. The aim of this systematic review and meta-analysis was to compare the efficacy of standard BQT versus concomitant therapy for *H. pylori* eradication in subjects naïve to treatment. (2) Methods: Online databases were searched for randomized controlled trials. We pooled risk ratio (RR) of individual studies for dichotomous outcomes using a random-effect model. (3) Results: Six studies with 1810 adults were included. Overall intention-to-treat (ITT) eradication rate was 87.4% with BQT and 85.2% with concomitant therapy (RR 1.01, 95%CI:0.94–1.07). Subgroup analysis of five Asian studies showed a small but significant superiority of BQT over concomitant therapy (87.5% vs. 84.5%; RR 1.04, 95%CI:1.01–1.08). Pooling four studies at low risk of bias yielded a similar result (88.2% vs. 84.5%; RR 1.05, 95%CI:1.01–1.09). There was no difference between the regimens in the frequency of adverse events (RR = 0.97, 95%CI:0.79–1.2). (4) Conclusions: The efficacy of BQT seems to be similar to concomitant therapy, with similar side effect profile. However, BQT showed a small but significant benefit over concomitant therapy in Asian populations and in studies at low risk of bias.

## 1. Introduction

*Helicobacter pylori* (*H. pylori*) infects more than half of adults globally [1] and is associated with gastric diseases, including dyspepsia, peptic ulcer (PU), and malignancies, such as gastric cancer and gastric mucosa-associated tissue lymphoma (MALT), and extra-gastric diseases [2,3]. There is unanimous consensus that all individuals with *H. pylori* infection should be offered an effective eradication therapy. The increasing resistance to antibiotics, in particular clarithromycin and metronidazole, is currently the main issue in the treatment of *H. pylori* infection [4]. Two quadruple regimens—the standard bismuth quadruple therapy and the non-bismuth quadruple (concomitant) therapy—have been proposed for the empirical first-line treatment of *H. pylori* infection in order to overcome the issue of antibiotic resistance [5]. The standard bismuth quadruple therapy is a complex regimen including proton pump inhibitor (PPI), bismuth salt, tetracycline, and metronidazole [6]; in order to overcome the complexity of bismuth quadruple therapy, a “three-in-one” capsule containing bismuth, tetracycline and metronidazole (Pylera^®^, Allergan, Inc., Dublin, Ireland) has been recently introduced in several countries [7]. The bismuth quadruple therapy is a “strong weapon” for clarithromycin and metronidazole resistance as this regimen does not contain clarithromycin, and metronidazole resistance is likely to be overcome by the synergism of metronidazole with bismuth. Bismuth quadruple therapy has the advantage to be effective against *H. pylori* strains with either single- or dual clarithromycin and metronidazole resistance [8], but its limited by its complexity and the high pill burden (14 pills per day). Concomitant therapy is a more recent regimen that consists of PPI, clarithromycin, amoxicillin, and metronidazole given all together. This regimen has the advantage to be less complex with a lower pill burden (8 pills per day) than bismuth quadruple therapy and to overcome the issue of single resistance to clarithromycin or metronidazole. However, concomitant therapy fails against *H. pylori* strains with dual clarithromycin and metronidazole resistance [8]. In addition, this regimen exposes patients to at least one unnecessary antibiotic which may contribute to increasing global antimicrobial resistance.

Although both quadruple regimens showed a good therapeutic performance reaching cure rates of 90% in non-randomized, real-life studies [9,10,11,12], bismuth quadruple therapy is the recommended first line-treatment for *H. pylori* infection, while concomitant therapy is considered as an alternative [13,14,15].

However, whether standard bismuth quadruple therapy is superior to concomitant therapy for *H. pylori* eradication is still unclear. Few randomized controlled trials (RCTs), most of them with small sample sizes, compared these two quadruple regimens with conflicting results. 

The aim of our study was to perform a systematic review and meta-analysis of randomized controlled trials to compare the efficacy of standard bismuth quadruple therapy versus concomitant therapy for the eradication of *H. pylori* in subjects naïve to treatment. 

## 2. Materials and Methods

We performed a systematic review and meta-analysis following the Cochrane recommendations for Systematic Reviews of Interventions [16] and the PRISMA statement, and registered the protocol in the PROSPERO International Registry (CRD42023415124).

### 2.1. Search Strategy and Study Selection

We searched MEDLINE via PubMed, Embase, the Cochrane Library, and Scopus databases up to 30 June 2022. The electronic search of the literature was carried out using the following keywords: “Helicobacter” or “*Helicobacter pylori*”, “quadruple therapy”, “bismuth quadruple therapy”, “Pylera”, “non-bismuth quadruple therapy” and “concomitant therapy”. The search strategies are reported in Supplementary Materials S1. We also searched electronically and by hand abstracts of the conferences’ proceedings of the International Workshop on *Helicobacter pylori* and Microbiota, United European Gastroenterology Week, American Digestive Disease Week, and Asian Pacific Digestive Week for the same period. There were no restrictions on language and publication status. We obtained a translation of any non-English article.

Two authors (E.D. and A.C.) independently performed the initial selection based on titles and abstracts and carried out a full-text assessment of potentially relevant publications; any disagreement was resolved by a third reviewer (R.M.Z.). 

For inclusion in the review, we selected studies if they met the following pre-specified criteria: randomized controlled trials comparing the efficacy of bismuth quadruple therapy versus concomitant therapy for the eradication of *H. pylori* infection in naïve subjects using the standardized regimens currently recommended by international guidelines [13,14,15]. Bismuth quadruple therapy must consist of PPI plus bismuth salt, tetracycline, and metronidazole or the three-in-one single capsule Pylera^®^, while the concomitant therapy must consist of PPI plus amoxicillin, clarithromycin, and metronidazole/tinidazole. Both bismuth quadruple and concomitant therapies must last at least 10 days. The study population may include adults or children diagnosed as *H. pylori* positive by at least one of the following tests: histology, rapid urease test, culture, urea breath test, or stool antigen test. The eradication of *H. pylori* should be confirmed by histology, urea breath test, or stool antigen test at least 4 weeks after the end of therapy. We excluded studies that did not meet the inclusion criteria (i.e., studies that used non-standardized-antibiotics different from those specified above—bismuth quadruple or concomitant therapies), or whether essential information was missing and could not be obtained by the authors. 

### 2.2. Data Extraction 

Two authors (E.D. and A.C.) independently extracted the following data from each study: year of publication, country, age and gender of participants, medical condition including peptic ulcer, non-ulcer dyspepsia, or other, diagnostic tests for *H. pylori* infection and assessment of eradication, number of participants in each treatment group, name, dose and timing of administration of PPI and antibiotics, duration of eradication treatment, eradication rates per treatment regimen, whether antimicrobial sensitivity and resistance were tested before and after treatment, incidence, type and severity of adverse events (AEs) and compliance.

### 2.3. Assessing the Risk of Bias 

Two authors (R.M.Z. and E.D.) independently assessed the risk of bias in studies using Version 2 of the Cochrane risk-of-bias tool for randomized trials (Rob 2) (Supplementary Materials S2) [17]. In particular, we evaluated the presence of potential bias in the following five domains: randomization process, deviations from the intended interventions, missing outcome data, measurement of the outcome, and selection of the reported result. The risk of bias judgments for each domain are “low risk of bias”, “some concerns” or “high risk of bias”. The risk of bias judgments are based on the answers to signalling questions according to an algorithm that maps responses to signalling questions to a final judgment (Supplementary Materials S2) [17]. The overall risk of bias for each study is based on the following criteria: “low risk of bias” if the study is judged at “low risk of bias” for all domains; “some concerns” if the study is judged to raise “some concerns” in at least one domain, but not to be at “high risk of bias” for any domain; “high risk of bias” if the study is judged to be at “high risk of bias” in at least one domain or to have “some concerns” for multiple domains [17]. Any disagreements were resolved by a third reviewer (F.B.).

### 2.4. Statistical Analysis

The primary outcome was the eradication rate of *H. pylori* according to the intention-to-treat analysis (ITT). The secondary outcome was the incidence of adverse events (AEs). 

We compared dichotomous outcomes of individual studies using the risk ratio (RR) with a 95% confidence interval (CI). We combined the RRs of individual studies in a pooled RR using a random-effect model. We used the Peto’s odds ratio (OR) method for outcomes with no events in one or both arms in individual studies [18].

Heterogeneity across the studies was assessed using the Q test and the I^2^ statistic; the I2 statistic was defined as low (<25%), moderate (25–50%), and high (>50%) [19]. For the primary outcome, we planned to perform subgroup analyses based on the geographic region (Asian vs. non-Asian studies), the duration of treatment (10 days vs. 14 days) and the risk of bias in the study (“low risk of bias” vs. “some concern” or “high risk of bias”). We defined as Asian studies those RCTs that were carried-out either in Asia or in countries with the majority of territories located in Asia. 

We investigated for publication bias by Egger test and funnel plot if at least 10 studies were included in the meta-analysis [18,20]. All the analyses were performed using STATA 16 (Stata Corp., College Station, TX, USA).

## 3. Results

**Figure 1:**
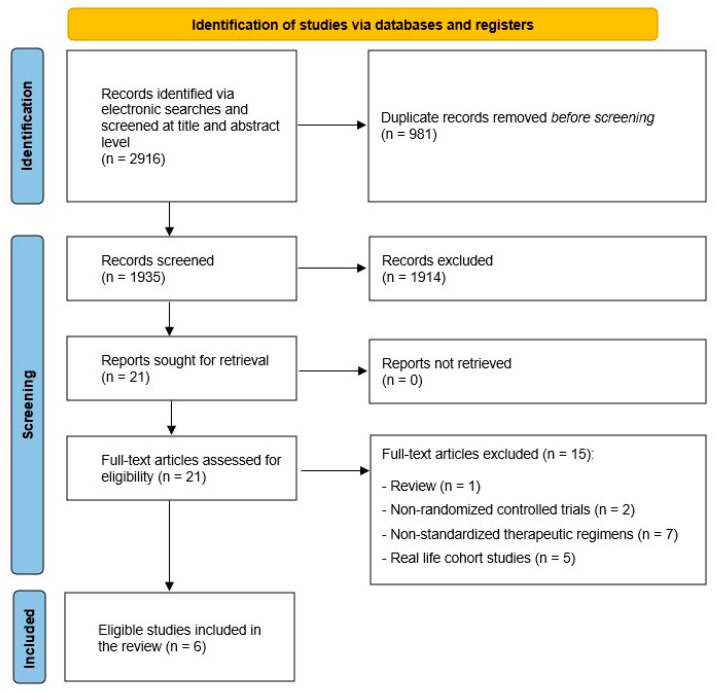
Flowchart of systematic literature search and study selection.

The electronic search identified 1463 records after duplicates were removed, of which 21 full-text articles were assessed for eligibility. Of the 21 articles, 6 met the criteria for inclusion in the review [8,21,22,23,24,25]. All studies were published in English, except one publication [25] in Russian that was translated. Figure 1 shows the flow chart of the selection process and the reasons for study exclusion. 

### 3.1. Study Characteristics

The six included studies involved adult subjects with mean age varying from 37 [21] to 58 [24] years and a proportion of men from 42% [22] to 53% [8]. The total sample size ranged from 70 [22] to 1080 [8] participants. Five studies were conducted in Asia (two in Turkey [21,22], one in Taiwan [8], one in Korea [24] and one in Russia [25]) and one in Europe (Italy) [23]. Except for two multi-centre trials [8,23], all studies were carried out in a single centre. Three studies enrolled patients with either non-ulcer dyspepsia or peptic ulcer [21,23,24], one with non-ulcer dyspepsia, peptic ulcer or asymptomatic subjects [8], one with only non-ulcer dyspepsia [22] and one with only peptic ulcer [25]. Table 1 shows the characteristics of the included studies.

The duration of both treatments was 10 days in four studies [8,21,23,25] and 14 days in two studies [22,24]. In all studies, the bismuth quadruple therapy included bismuth sub-citrate, tetracycline, and metronidazole with different dosages; antibiotics were taken separately in all studies, except from one [23] that used the “three-in-one” single capsule Pylera^®^. In all studies concomitant therapy consisted of clarithromycin, amoxicillin, and metronidazole, except for one [23] that used tinidazole instead of metronidazole. Both bismuth quadruple therapy and concomitant therapy included different PPIs; two studies used rabeprazole [21,22], two studies lansoprazole [8,24], one study omeprazole [25], and one study esomeprazole [23]. All studies used a standard dose of PPI (rabeprazole 20 mg, lansoprazole 30 mg, omeprazole 20 mg, and esomeprazole 20 mg) twice daily, except for the study by Kefeli [21] that used high-dose PPI (rabeprazole 40 mg) twice daily. Details on the bismuth quadruple therapy and concomitant therapy are reported in Supplementary Table S1. Only the study by Liou [8] performed prior antibiotic susceptibility testing and reported eradication rates by antibiotic resistance. 

### 3.2. Risk of Bias

**Figure 2:**
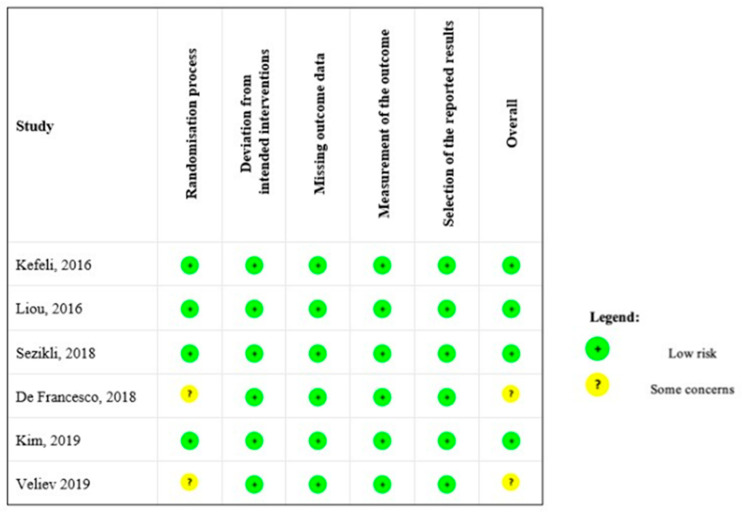
Risk of bias of included studies. Kefeli 2016 [21], Liou 2016 [8], Sezikli 2018 [22], De Francesco 2018 [23], Kim 2019 [24], Veliev 2019 [25].

Four RCTs were judged to be at “low risk of bias” in all domains [8,21,22,24], while two studies [23,25] raised “some concerns” in the “randomization process” domain. In these two studies, there was no information on how the random sequence was generated and whether the allocation sequence was concealed at randomization. Figure 2 and Supplementary Materials S2 show the results of the assessment of the risk of bias in the included trials.

### 3.3. Eradication of H. pylori Infection

**Figure 3:**
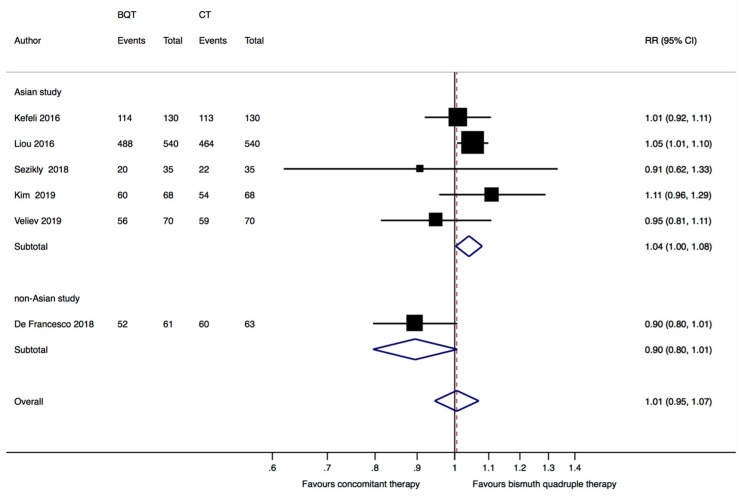
Forest plot of risk ratio (RR) in the intention to treat eradication rate between standard bismuth quadruple therapy (BQT) and concomitant therapy (CT) by geographic region (Asian study vs. non Asian study). CI: confidence interval. Kefeli 2016 [21], Liou 2016 [8], Sezikli 2018 [22], De Francesco 2018 [23], Kim 2019 [24], Veliev 2019 [25].

The six included studies reported data from a total of 1810 subjects, of whom 904 were randomized to receive the standard bismuth quadruple therapy and 906 the concomitant therapy. The pooled analysis of the six studies showed no significant difference between bismuth quadruple therapy and concomitant therapy in the *H. pylori* eradication rates (RR = 1.01, 95%CI: 0.94–1.07) (Figure 3). The overall ITT eradication rate was 87.4% (709/904) with bismuth quadruple therapy and 85.2% (772/906) with concomitant therapy. There was moderate heterogeneity among the studies (I^2^ = 44.1%).

The subgroup analysis of the five Asian studies [8,21,22,24,25] showed a small but significant advantage for bismuth quadruple therapy over concomitant therapy, with an eradication rate of 87.5% (738/843) in the bismuth quadruple therapy group and 84.5% (712/843) in the concomitant therapy group (RR = 1.04, 95%CI: 1.01–1.08, *p* = 0.03, I^2^ = 0%); the European study [23] reported similar eradication rates in both treatment arms (RR = 0.9, 95%CI: 0.8–1.01) (Figure 3).

**Figure 4:**
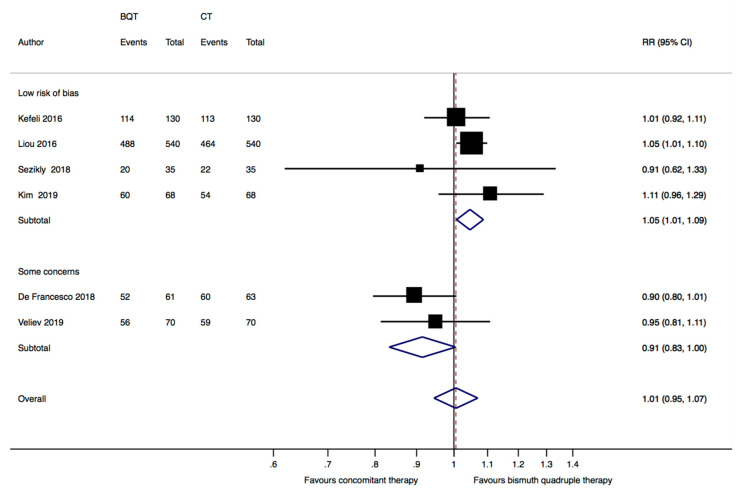
Forest plot of risk ratio (RR) in the intention to treat eradication rate between standard bismuth quadruple therapy (BQT) and concomitant therapy (CT) by overall risk of bias (“low risk of bias” in all domains vs. “some concerns” in at least one domain). CI: confidence interval. Kefeli 2016 [21], Liou 2016 [8], Sezikli 2018 [22], De Francesco 2018 [23], Kim 2019 [24], Veliev 2019 [25].

The subgroup analysis of the four studies [8,21,22,24] at low risk of bias also yielded a significant higher eradication rate with bismuth quadruple therapy (88.2%, 682/773) in comparison to concomitant therapy (84.5%, 653/773) (RR = 1.05, 95%CI: 1.01–1.09, *p* = 0.02, I^2^ = 0%). Pooling the two studies [23,25] with “some concerns” in the “randomization process” domain showed no difference between the two groups (RR = 0.91, 95%CI: 0.83–1.01, I^2^ = 44.1%) (Figure 4).

The subgroup analysis based on the duration of therapy did not show significant differences between bismuth quadruple therapy and concomitant therapy in the ITT eradication rate at 10 days and 14 days. Pooling four trials [8,21,23,25] comparing 10-day bismuth quadruple therapy versus 10-day concomitant therapy yielded a risk ratio of 0.99 (95%CI: 0.92–1.07), and pooling two studies [22,24] comparing 14-day bismuth quadruple versus 14-day concomitant therapy resulted in a risk ratio of 1.08 (95%CI: 0.92–1.26) (Supplementary Figure S1).

One study [8] reported eradication rates by antibiotic resistance showing that bismuth quadruple therapy was more efficacy than concomitant therapy in subjects with single clarithromycin resistance (eradication rate: 89% vs. 72%) or dual clarithromycin and metronidazole resistance (94% vs. 59%).

### 3.4. Adverse Events and Compliance

**Figure 5:**
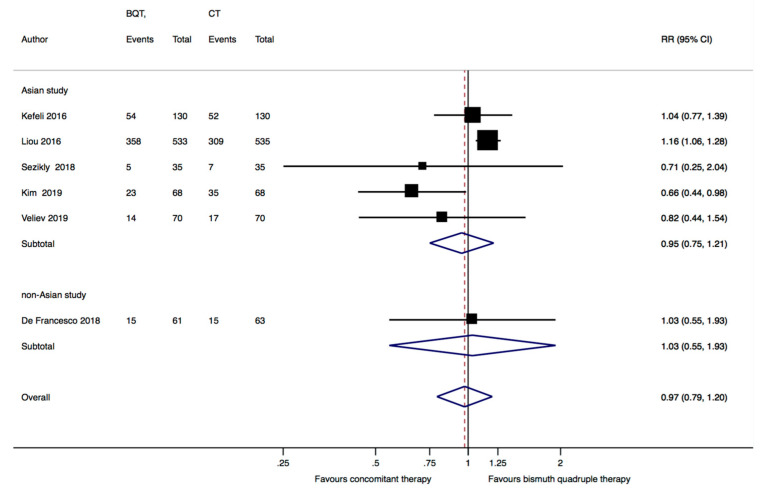
Forest plot of risk ratio (RR) in the frequency of adverse events treatment-related between standard bismuth quadruple therapy (BQT) and concomitant therapy (CT) by geopraphic region (Asian study vs. non Asian study). CI: confidence interval. Kefeli 2016 [21], Liou 2016 [8], Sezikli 2018 [22], De Francesco 2018 [23], Kim 2019 [24], Veliev 2019 [25].

Pooling data from the six studies, the overall incidence of AEs was 52.3% (469/897) in patients treated with bismuth quadruple therapy and 48.3% (435/901) in those treated with concomitant therapy, with no statistical difference between the two groups (RR = 0.97, 95%CI: 0.79–1.2, I^2^ = 47.1%) (Figure 5). The overall rate of patients who stopped drugs because of severe AEs was similar for the two regimens: 6.5% (58/897) for bismuth quadruple therapy and 4.5% (41/901) for concomitant therapy (Peto’s odds ratio = 1.21, 95%CI: 0.92–1.58).

Compliance was defined as “good” if participants took at least 80% of the drugs in four studies [8,21,22,25] and 90% of drugs in two studies [23,24]. Five studies [8,21,22,23,25] reported “good compliance” in more than 90% of participants and one study [24] in more than 80% of participants, with no significant differences between the study groups.

## 4. Discussion

Our meta-analysis of RCTs showed that in adults the efficacy of standard bismuth quadruple therapy is similar to concomitant therapy, regardless of the duration of therapy, in the first-line treatment of *H. pylori* infection. However, this result is not robust as subgroup analyses yielded a small but significant superiority of bismuth quadruple therapy over concomitant therapy in Asian countries and in studies at low risk of bias. Incidence of adverse events and compliance were similar between the two regimens suggesting that bismuth quadruple therapy is as tolerated and safe as concomitant therapy.

To our knowledge, this is the first systematic review with meta-analysis to compare the efficacy of bismuth quadruple therapy versus concomitant therapy using (a) a comprehensive literature search, (b) an appropriate tool for assessing the risk of bias in randomized trials (the Rob 2), and (c) standardized regimens recommended by international guidelines [13,14,15], in terms of antibiotic composition and duration of treatment.

A recent meta-analysis by Guo et al. [26] comparing bismuth quadruple therapy and concomitant therapy included non-standardized and unoptimized regimens. Indeed, in this study, the “bismuth-containing quadruple treatment” was defined as a “PPI and bismuth plus two kinds of antibiotics” and the “concomitant treatment” as a “PPI plus three kinds of antibiotics”. There is no doubt that the inclusion of only standardized and optimized regimens, currently recommended by guidelines and used in clinical practice, is the more adequate and less biased approach for comparing bismuth quadruple therapy and concomitant therapy [27]. In addition, the systematic review by Guo et al. did not include the study by Veliev [25], that was included in our meta-analysis.

The meta-analysis by Guo et al. [26] reported comparable ITT eradication rates between bismuth quadruple therapy and concomitant therapy (OR: 1.14, 95%CI: 0.94–1.38). We reported a similar result, but differently from Guo et al. we found a small but significant advantage for bismuth quadruple therapy over concomitant therapy in Asian countries. It is well known that geographic regions may affect the relative efficacy of treatments. One of the most important factors that can affect the outcome of *H. pylori* therapy is the prevalence of antimicrobial resistance as well as other host factors, such as those affecting metabolism of PPIs (e.g., CYP2C19 polymorphisms) and acid secretion (e.g., race and body mass index, etc.). Indeed, there is a substantial difference between Western and Asian populations in terms of the prevalence of single and dual clarithromycin and metronidazole resistance and of the proportion of subjects with slow PPI metabolizers, both higher in Asia than in Europe [27,28]. In particular, the high prevalence of dual clarithromycin and metronidazole resistance in Asian populations would explain the superiority of bismuth quadruple therapy over concomitant therapy, that has a lower efficacy towards dual clarithromycin and metronidazole resistance. On the other hand, the European trial [23] showed similar eradication rates between bismuth quadruple therapy and concomitant therapy, in line with another non-randomized trial carried out in Spain [12], likely due to the low prevalence (<15%) of dual clarithromycin and metronidazole resistance in Western countries [29].

The meta-analysis by Guo et al. [26] reported a similar frequency of AEs with bismuth quadruple therapy and concomitant therapy (36.4% vs. 34.4%, respectively; OR: 1.02, 95%CI: 0.74–1.40). We reported a similar result, but in our study the frequency of AEs was higher than in the study by Guo et al. for both bismuth quadruple therapy and concomitant therapy (52.3% and 48.3.%, respectively).

A strength of this study is the exhaustive search of literature, with no restrictions for language or type of publications. As less than 10 studies are included in the meta-analysis, we are not able to test for publication bias and assess the likely impact of unpublished studies on our results; however, the included studies in this systematic review represent very likely the majority on this topic. Another strength of this study is that systematic review, assessment of risk of bias in RCTs and analyses were performed following the Cochrane recommendations for Systematic Reviews of Interventions [16].

A weakness of this study is that only six RCTs were included in the pooled analysis. Another weakness is that the result of our meta-analysis was not robust after the exclusion of the two studies [23,25] that raised “some concern” in the “randomisation process” due to the lack of information on the random sequence generation and allocation sequence concealment at randomization. In fact, pooling the four RCTs at low risk of bias resulted in a small by significantly higher eradication rate with bismuth quadruple therapy compared to concomitant therapy. It is well known that issues in the randomisation process may bias the estimated effect of treatments by introducing known and unknown confounding factors [30]. Thus, we cannot exclude that methodological diversity among studies may have led to differences in the intervention effects making null the small but statistically significant advantage of bismuth quadruple therapy over concomitant therapy that was showed by RCTs at low risk of bias. However, although studies at low risk of bias provide a stronger contribution to the certainty of evidence, their number in our analysis was very small.

Another weakness is that only one trial [8] provided data on *H. pylori* antimicrobial resistance. The remaining trials were conducted in high clarithromycin-resistance areas, but eradication rates according to the antibiotic susceptibility of *H. pylori* were not available. Finally, none of studies reported eradication rates based on the underlying disease, peptic ulcer, and non-ulcer dyspepsia mainly.

In conclusion, our meta-analysis shows that the efficacy of standard bismuth quadruple therapy is similar to concomitant therapy in the first line treatment of *H. pylori* infection, with a similar safety profile. However, sub-group analyses suggest that this finding is not robust as bismuth quadruple therapy has a small but significant benefit over concomitant therapy in Asia populations and in studies at low risk of bias.

Differently from non-randomized studies [9,10,11,12], in our meta-analysis neither regimen achieved the optimal cure rate of 90%. However, the overall ITT cure rate was 87.4% for bismuth quadruple therapy and 85.2% for concomitant therapy, which is considered “borderline acceptable” in clinical practice, being between 85% and 89% [27].

Our findings still support guidelines recommendations to prefer bismuth quadruple therapy over concomitant therapy as first-line treatment of *H. pylori* infection. Bismuth quadruple therapy avoids the exposure to unnecessary antibiotics, and, in addition, yields a slightly greater chance of *H. pylori* eradication in Asian countries [28] and in populations with similar high clarithromycin and metronidazole resistance rates. If bismuth, tetracycline, or Pylera^®^ are not available concomitant therapy has adequate efficacy to be considered as a valid alternative.

However, well-designed RCTs with a large sample size are needed to confirm our findings, especially in Europe and America. These studies should possibly also provide eradication rates according to the antimicrobial susceptibility of *H. pylori* and by groups of people with different underlying diseases, as these data are scarce or not available.

## Figures and Tables

**Table 1:** Characteristics of the included studies.

Study, Year	Country	Total Patients, n.	Age Mean, Year	Males n, (%)	Medical Condition	Tests for *H. pylori* Diagnosis	Test for *H. pylori* Eradication	Eradication Rate (ITT)		Side Effects	
								BQT n. (%)	CT n. (%)	BQT n. (%)	CT n. (%)
Kefeli, 2016 [21]	Turkey	260	37.3	138 (53.1)	PU, NUD	Histology	UBT after ≥ 6 weeks	114/130 (87.7)	113/130 (86.9)	54/130 (41.5)	52/130 (40)
Liou, 2016 [8]	Taiwan	1080	53.3	534 (49.4)	PU, NUD, asymptomatic individuals	2 out of 4: histology, RUT, culture, serology	UBT after > 6 weeks	488/540 (90.4)	464/540 (85.9)	358/533 (67.2)	309/535 (57.8)
Sezikli, 2018 [22]	Turkey	70	41.8	30 (42.9)	NUD	Histology	UBT after 6–8 weeks	20/35 (57.1)	22/35 (62.8)	5/35 (14.3)	7/35 (20)
De Francesco, 2018 [23]	Italy	124	53.6	55 (44.3)	PU, NUD	Histology and RUT	UBT after 6–8 weeks	52/61 (85.2)	60/63 (95.2)	15/61 (24.6)	15/63 (23.8)
Kim, 2019 [24]	Korea	136	58.7	72 (52.9)	PU, NUD	Histology or RUT	UBT after ≥ 4 weeks	60/68 (88.2)	54/68 (79.4)	23/68 (33.8)	35/68 (51.5)
Veliev 2019 [25]	Russia	140	N/A	N/A	PU	RUT	UBT after ≥ 4 weeks	56/70 (80)	59/70 (84.2)	14/70 (20)	17/70 (24.2)

PU: peptic ulcer; NUD: non-ulcer dyspepsia; RUT: rapid urease test; UBT: urea breath test; BQT: bismuth quadruple therapy; CT: concomitant therapy; ITT: intention-to-treat.

## Data Availability

The data presented in this study are available on reasonable request from the corresponding author.

## Supplementary Materials

The following supporting information can be downloaded at: https://www.mdpi.com/article/10.3390/jcm12093258/s1, Supplementary Materials S1—Electronic Search Strategy; Supplementary Materials S2—Answers to signalling questions in the 5 domains of the risk-of-bias tool for randomized trials (RoB2) for each included study. Table S1—Details of bismuth quadruple therapy and concomitant therapy. Figure S1. Forest plot of risk ratio (RR) in the intention to treat eradication rate between standard bismuth quadruple therapy (BQT) and concomitant therapy (CT) by duration of treatment (10 days vs. 14 days). Kefeli 2016 [21], Liou 2016 [8], Sezikli 2018 [22], De Francesco 2018 [23], Kim 2019 [24], Veliev 2019 [25].
