# Supplementary material for: Standard Bismuth Quadruple Therapy versus Concomitant Therapy for the First-Line Treatment of Helicobacter pylori Infection: A Systematic Review and Meta-Analysis of Randomized Controlled Trials
Source: J Clin Med. 2023 May 3;12(9):3258. doi: 10.3390/jcm12093258 (PMC10179629; doi:10.3390/jcm12093258)
Supplement: Supplementary file 1 [file jcm-12-03258-s001.zip › jcm-2299001-supplementary/jcm-2299001-supplementary.pdf]

Journal of
Clinical Medicine

MDPI

Supplementary Materials S1. Electronic Search Strategy (30.06.2022)

MEDLINE via PubMed (n. 1040)

("Helicobacter" OR "Helicobacter pylori" or "Helicobacter Infections" or "Helicobacter Infection" OR campylobacter or hp or pylori or pyloridis) and (quadruple or concomitant or non-bismuth) and (bismuth or pylera)

SCOPUS (n. 1151)

TITLE-ABS-KEY ("Helicobacter" OR "Helicobacter pylori" OR "Helicobacter Infections" OR "Helicobacter Infection" OR campylobacter OR pylori OR pyloridis) AND TITLE-ABS-KEY (quadruple OR concomitant OR non-bismuth) AND TITLE-ABS-KEY (bismuth OR pylera)

Ovid Embase (n. 393)

#1 ('helicobacter infection'/exp OR 'helicobacter infection':ti,ab OR 'helicobacter pylori':ti,ab OR 'h. pylori':ti,ab)

#2 ('non bismuth quadruple':ti,ab OR concomitant:ti,ab)

#3 (bismuth:ti,ab OR pylera:ti,ab)

#1 AND #2 AND #3

COCHRANE Library (n. 332)

#1 ("Helicobacter" OR "Helicobacter pylori" OR "hp" or "H. pylori"):ti,ab,kw

#2 ("quadruple" OR "concomitant" OR "non-bismuth"):ti,ab,kw

#3 ("bismuth" OR "pylera"):ti,ab,kw

#4 ("randomized controlled trial" OR "randomized trial" OR "RCT"):ti,ab,kw)

#1 AND #2 AND #3 AND #4

Supplementary Materials S2. Answers to signalling questions in the 5 domains of the risk-of-bias tool for randomized trials (RoB2) for each included study.

Domain 1. Bias arising from the randomisation process			
	**1.1 Was the allocation sequence random?**	**1.2 Was the allocation sequence concealed until participants were enrolled and assigned to intervention?**	1.3. Did baseline differences between intervention groups suggest a problem with the randomization process?
Kefeli, 2016	Y	PY	PN
Liou, 2016	Y	Y	N
Sezikli, 2018,	Y	Y	PN
De Francesco, 2018	NI	NI	N
Kim, 2019	Y	PY	N
Veliev, 2019	NI	NI	NI

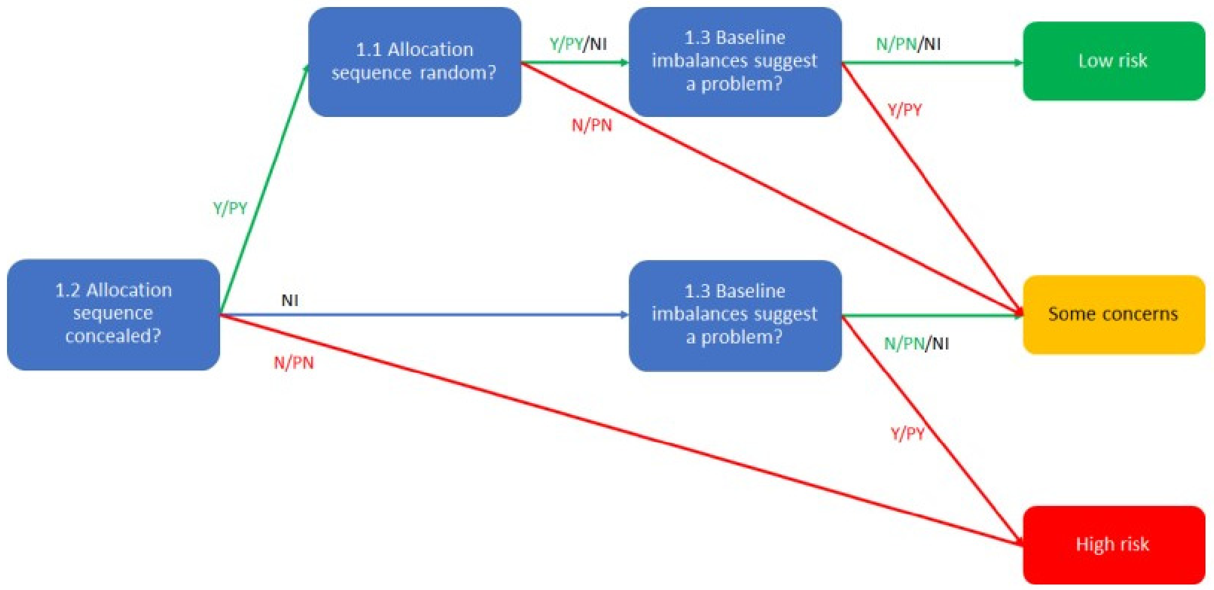

Domain 2. Risk of bias due to deviations from the intended interventions							
	2.1. Were participants aware of their assigned intervention during the trial?	2.2. Were carers and people delivering the interventions aware of participants' assigned intervention during the trial?	2.3. If Y/PY/NI to 2.1 or 2.2: Were there deviations from the intended intervention that arose because of the trial context?	2.4. If Y/PY to 2.3: Were these deviations likely to have affected the outcome?	2.5. If Y/PY/NI to 2.4: Were these deviations from intended intervention balanced between groups?	2.6. Was an appropriate analysis used to estimate the effect of assignment to intervention?	2.7. If N/PN/NI to 2.6: Was there potential for a substantial impact (on the result) of the failure to analyse participants in the group to which they were randomized?
Kefeli, 2016	Y	Y	N	-	-	Y	-
Liou, 2016	Y	N	N	-	-	Y	-
Sezikli, 2018,	Y	Y	N	-	-	Y	-
De Francesco, 2018	Y	Y	N	-	-	Y	-
Kim, 2019	Y	Y	N	-	-	Y	-
Veliev, 2019	Y	Y	N	-	-	Y	-

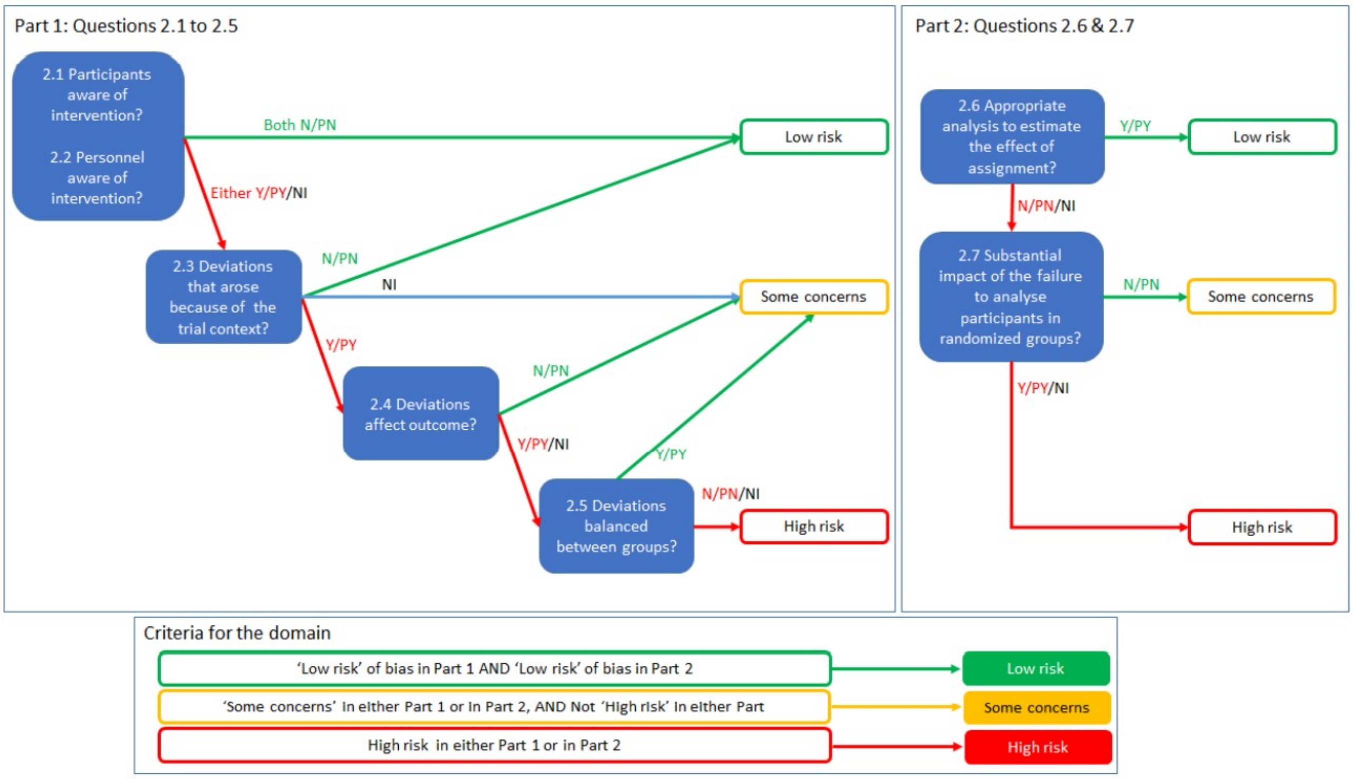

**Domain 3. Risk of bias due to missing outcome data**				
	3.1. Were data for this outcome available for all, or nearly all, participants randomized?	3.2. If N/PN/NI to 3.1: Is there evidence that the result was not biased by missing outcome data?	3.3. If N/PN to 3.2: Could missingness in the outcome depend on its true value?	3.4. If Y/PY/NI to 3.3: Is it likely that missingness in the outcome depended on its true value?
Kefeli, 2016	Y	-	-	-
Liou, 2016	Y	-	-	-
Sezikli, 2018,	Y	-	-	-
De Francesco, 2018	Y	-	-	-
Kim, 2019	Y	-	-	-
Veliev, 2019	Y	-	-	-

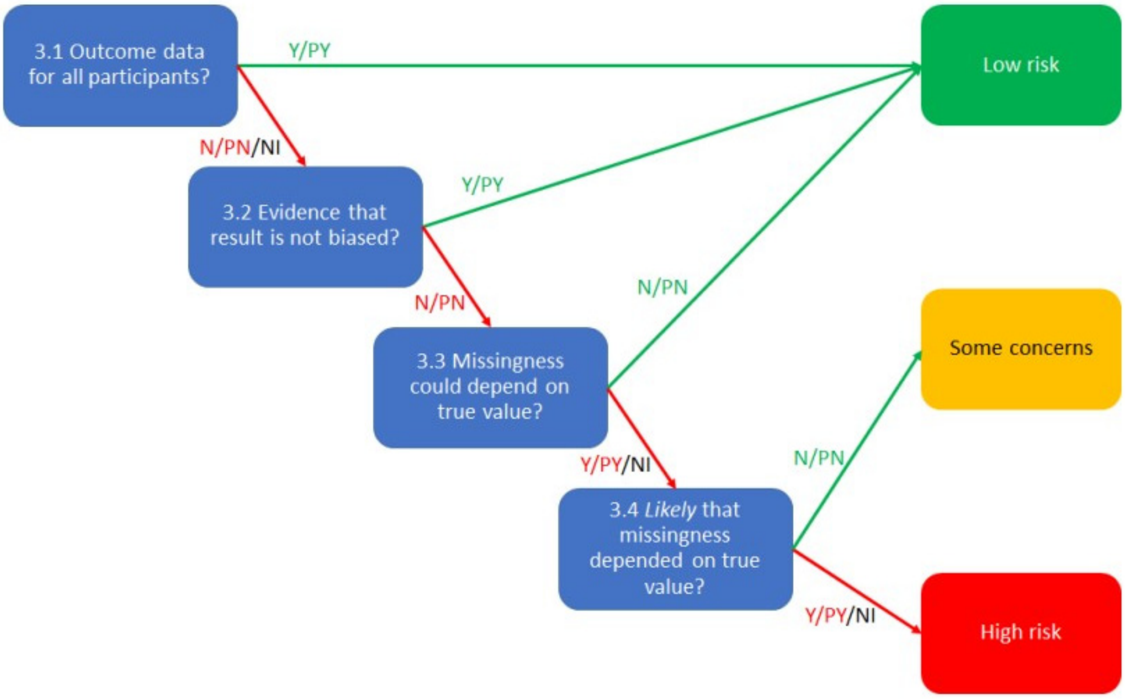

Domain 4. Risk of bias in measurement of the outcome					
	4.1. Was the method of measuring the outcome inappropriate?	4.2. Could measurement or ascertainment of the outcome have differed between intervention groups?	4.3. Were outcome assessors aware of the intervention received by study participants?	4.4. Could assessment of the outcome have been influenced by knowledge of intervention received?	4..5 If Y/PY/NI to 4.4: Is it likely that assessment of the outcome was influenced by knowledge of intervention received?
Kefeli, 2016	N	N	NI	N	-
Liou, 2016	N	N	NI	N	-
Sezikli, 2018,	N	N	NI	N	-
De Francesco, 2018	N	N	NI	N	-
Kim, 2019	N	N	NI	N	-
Veliev, 2019	N	N	NI	N	-

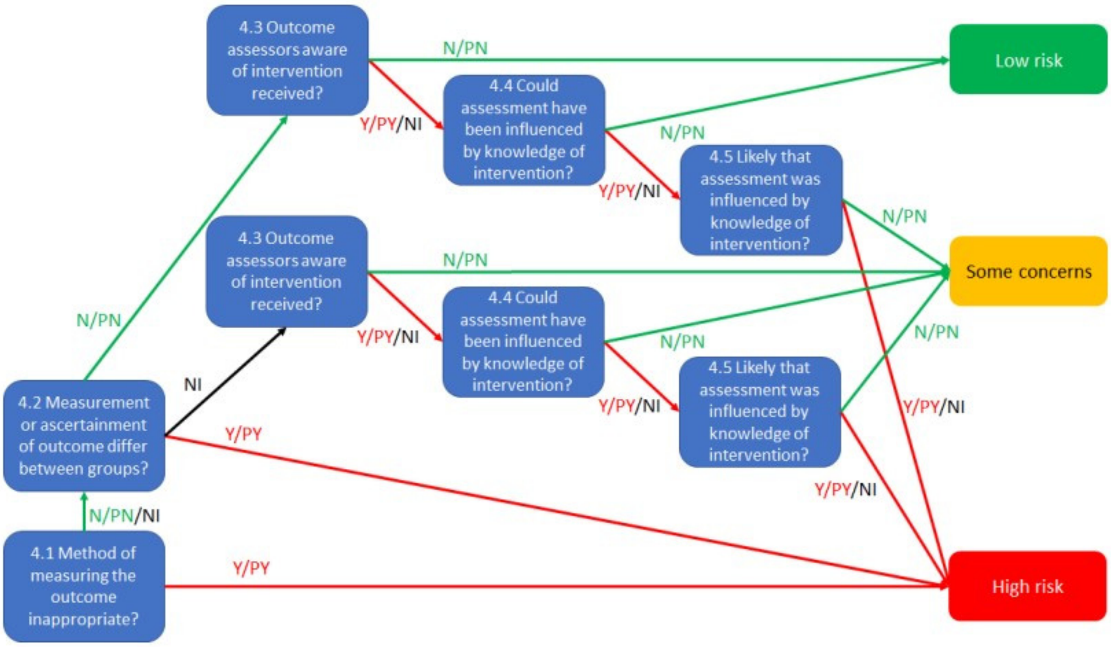

Domain 5. Risk of bias in selection of the reported results			
	5.1. Were the data that produced this result analysed in accordance with a pre-specified analysis plan that was finalized before unblinded outcome data were available for analysis?	5.2. Is the numerical result being assessed likely to have been selected, on the basis of the results, from multiple eligible outcome (e.g. scales, definition, time points) within the outcome domain?	5.3. Is the numerical result being assessed likely to have been selected, on the basis of the results, from multiple eligible analyses of the data?
Kefeli, 2016	Y	N	N
Liou, 2016	Y	N	N
Sezikli, 2018,	Y	N	N
De Francesco, 2018	Y	N	N
Kim, 2019	Y	N	N
Veliev, 2019	Y	N	N

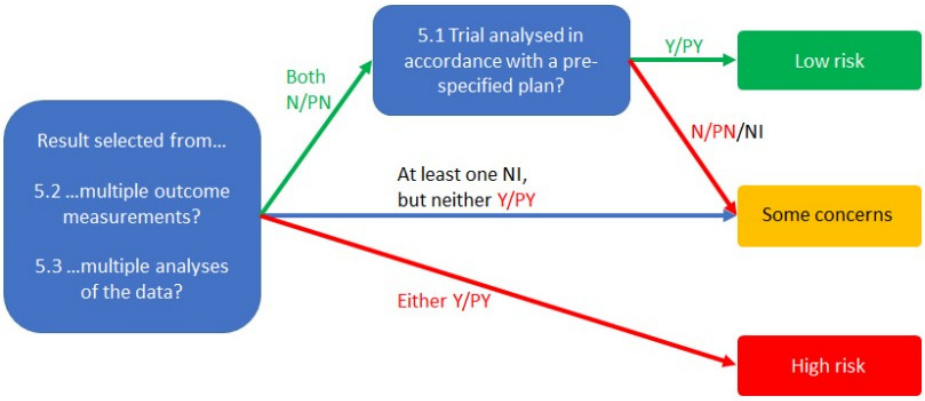

Table S1. Details of bismuth quadruple therapy and concomitant therapy.

	Bismuth quadruple therapy			Concomitant therapy		
**Study, Year**	**Regimen**	**Dosage**	**Duration (Days)**	**Regimen**	**Dosage**	**Duration (Days)**
Kefeli, 2016	Rabeprazole<br>Bismuth potassium citrate<br>Tetracycline<br>Metronidazole	40 mg b.i.d.<br>220 mg b.i.d.<br>500 mg t.i.d.<br>500 mg t.i.d	10	Rabeprazole<br>Amoxicillin<br>Clarithromycin<br>Metronidazole	40 mg b.i.d.<br>1000 mg b.i.d.<br>500 mg b.i.d.<br>500 mg t.i.d.	10
Liou, 2016	Lansoprazole<br>Bismuth tripotassium dicitrate<br>Tetracycline<br>Metronidazole	30 mg b.i.d.<br>300 mg q.i.d.<br>500 mg q.i.d.<br>500 mg t.i.d	10	Lansoprazole<br>Amoxicillin<br>Clarithromycin<br>Metronidazole	30 mg b.i.d.<br>1000 mg b.i.d.<br>500 mg b.i.d.<br>500 mg t.i.d.	10
Sezikli, 2018	Rabeprazole<br>Bismuth potassium citrate<br>Tetracycline<br>Metronidazole	20 mg b.i.d.<br>220 q.i.d.<br>500 mg q.i.d.<br>500 mg t.i.d	14	Rabeprazole<br>Amoxicillin<br>Clarithromycin<br>Metronidazole	20 mg b.i.d.<br>1000 mg b.i.d.<br>500 mg b.i.d.<br>500 mg t.i.d.	14
De Francesco, 2018	Esomeprazole<br>Bismuth subcitrate potassium<br>Tetracycline<br>Metronidazole	20 mg b.i.d.<br>420 mg q.i.d.<br>375 mg q.i.d.<br>375 mg q.i.d.	10	Esomeprazole<br>Amoxicillin<br>Clarithromycin<br>Tinidazole	20 mg b.i.d.<br>1000 mg b.i.d.<br>500 mg b.i.d.<br>500 mg b.i.d.	10
Kim, 2019	Lansoprazole<br>Bismuth tripotassium dicitrate<br>Tetracycline<br>Metronidazole	30 mg b.i.d.<br>600 mg b.i.d.<br>1000 mg b.i.d.<br>500 mg b.i.d	14	Lansoprazole<br>Amoxicillin<br>Clarithromycin<br>Metronidazole	30 mg b.i.d.<br>1000 mg b.i.d.<br>500 mg b.i.d.<br>500 mg t.i.d.	14
Veliev 2019	Omeprazole<br>Bismuth subcitrate potassium<br>Tetracycline<br>Metronidazole	20 mg b.i.d.<br>120 mg q.i.d.<br>500 mg q.i.d.<br>500 mg t.i.d	10	Omeprazole<br>Amoxicillin<br>Clarithromycin<br>Metronidazole	20 mg b.i.d.<br>1000 mg b.i.d.<br>500 mg b.i.d.<br>500 mg b.i.d.	10

SD: standard deviation; ITT: intention-to-treat; PP: per-protocol.

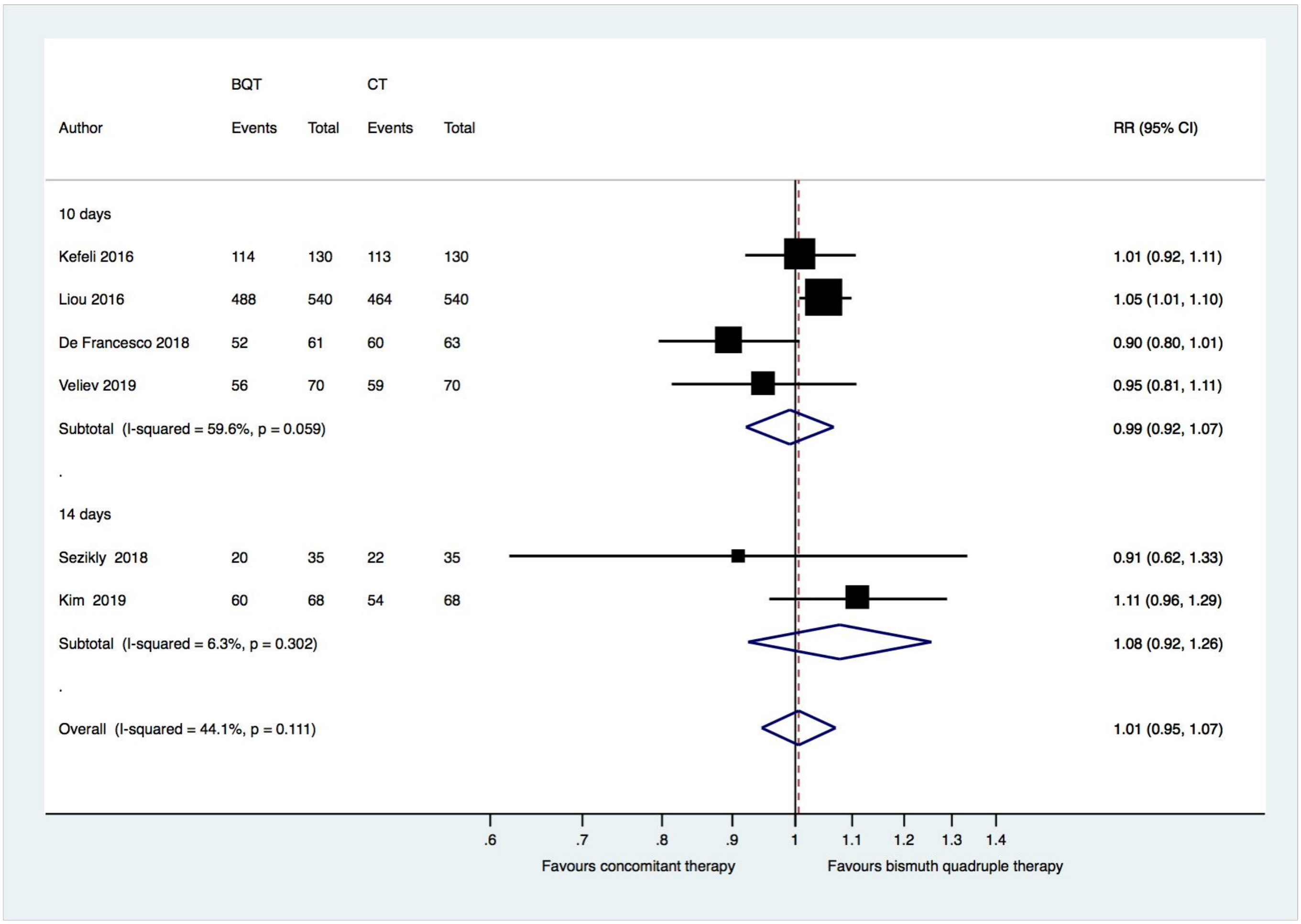

Figure S1. Forest plot of risk ratio (RR) in the intention to treat eradication rate between standard bismuth quadruple therapy (BQT) and concomitant therapy (CT) by duration of treatment (10 days vs 14 days). Kefeli 2016 [21], Liou 2016[8] Sezikli 2018 [22], De Francesco 2018 [23], Kim 2019 [24], Veliev 2019 [25].
